# Zwitterion Co-Polymer PEI-SBMA Nanofiltration Membrane Modified by Fast Second Interfacial Polymerization

**DOI:** 10.3390/polym12020269

**Published:** 2020-01-27

**Authors:** Yu-Hsuan Chiao, Tanmoy Patra, Micah Belle Marie Yap Ang, Shu-Ting Chen, Jorge Almodovar, Xianghong Qian, S. Ranil Wickramasinghe, Wei-Song Hung, Shu-Hsien Huang, Yung Chang, Juin-Yih Lai

**Affiliations:** 1 Graduate Institute of Applied Science and Technology, National Taiwan University of Science and Technology, Taipei 10607, Taiwan; ychiao@uark.edu (Y.-H.C.); jylai@mail.ntust.edu.tw (J.-Y.L.); 2 Ralph E. Martin Department of Chemical Engineering, University of Arkansas, Fayetteville, AR 72701, USA; sc068@uark.edu (S.-T.C.); jlalmodo@uark.edu (J.A.); 3 R&D Center for Membrane Technology and Department of Chemical Engineering, Chung Yuan University, Chung Li 32023, Taiwan; mbmyang@gmail.com (M.B.M.Y.A.); huangsh@niu.edu.tw (S.-H.H.); changyung0307@gmail.com (Y.C.); 4 Department of Biomedical Engineering, University of Arkansas, Fayetteville, AR 72701, USA; tanmoypatra29@gmail.com (T.P.); xqian@uark.edu (X.Q.); 5 Department of Chemical and Materials Engineering, National Ilan University, Yi-Lan 26047, Taiwan

**Keywords:** zwitterion polymer, nanofiltration, antifouling, second interfacial polymerization, PEI-SBMA

## Abstract

Nanofiltration membranes have evolved as a promising solution to tackle the clean water scarcity and wastewater treatment processes with their low energy requirement and environment friendly operating conditions. Thin film composite nanofiltration membranes with high permeability, and excellent antifouling and antibacterial properties are important component for wastewater treatment and clean drinking water production units. In the scope of this study, thin film composite nanofiltration membranes were fabricated using polyacrylonitrile (PAN) support and fast second interfacial polymerization modification methods by grafting polyethylene amine and zwitterionic sulfobutane methacrylate moieties. Chemical and physical alteration in structure of the membranes were characterized using methods like ATR-FTIR spectroscopy, XPS analysis, FESEM and AFM imaging. The effects of second interfacial polymerization to incorporate polyamide layer and ‘ion pair’ characteristics, in terms of water contact angle and surface charge analysis was investigated in correlation with nanofiltration performance. Furthermore, the membrane characteristics in terms of antifouling properties were evaluated using model protein foulants like bovine serum albumin and lysozyme. Antibacterial properties of the modified membranes were investigated using *E. coli* as model biofoulant. Overall, the effect of second interfacial polymerization without affecting the selectivity layer of nanofiltration membrane for their potential large-scale application was investigated in detail.

## 1. Introduction

Clean water production as per the ever-growing demand has become a major challenge in recent years with rapid population growth and extended droughts [1]. Nanofiltration (NF) is a low-cost pressure-driven membrane process which is considered as one such promising solution for the drinking as well as wastewater treatment to tackle the water crisis. In general, NF process has diverse application in liquid phase treatment such as desalination, food, pharmaceutical, solvent separation, biomass recovery, and oil/water separation [1,2,3,4,5,6]. The required pore size of the separation layer is in between reverse osmosis and ultrafiltration, as a result, NF has a lower demand for hydraulic pressure and results in higher multivalent ions rejection.

NF membranes were first reported back in the 1970s using aqueous monomer piperazine (PIP) and organic monomer trimesoyl chloride (TMC) via interfacial polymerization [7,8]. Apart from interfacial polymerization, several modifications have been used to create a selective layer on the support membrane such as dip-coating [9], layer-by-layer [10], interpenetrating polymer networks (IPN) [11], grafting [12], plasma [13], biocoating [14], and pressure assistant assembly [15]. Phase inversion methods have also been employed for the fabrication of mixed matrix NF membrane [16]. Among all the modification methods, the interfacial polymerization is considered as a fast and highly efficient method for preparation of the commercial thin-film composite (TFC) membranes. Using this method, the selectivity layer is generated at the interface between two liquid phases, and their functional moiety is tuned by monomer (type and concentration), time, temperature, and post-treatment [17]. Furthermore, aqueous or organic phase additives have been widely studied to tailor the structure and enhance the separation performance [4,18,19]. In general, the TFC membrane modifications can be divided into three methods in-situ, ‘grafting from’, and ‘grafting to’. Although In-situ is the most commonly used method, but the alteration in structure of selectivity layer leads to significant loss of rejection [2,20]. In general, ‘grafting from’ methods like atom transfer radical polymerization (ATRP) [6] and dopamine self-polymerization [21,22] provide precise control over the grafting density and chain length; however, the reaction time is long, and chemical consumption is high. In terms of commercialization these processes are highly unlikely unless specific application is required [20]. In case of the ‘grafting to’ method, the functionalized monomers or polymers are synthesized before grafting to the membrane surface, and the uniform chemical structure can be efficiently controlled. The fast second interfacial polymerization is a special type of method for modification between in situ and ‘grafting from’ method, with inherent advantages like short reaction time and uniform functional chain length. Moreover, the second interfacial polymerization can be easily integrated to existing production system, and hence, it is used very often in commercialized modification methods. However, the antifouling and antibacterial properties of such membranes have been a major bottleneck for their large-scale commercial application.

To enhance the antifouling property of NF membranes for processing severely polluted feed steam such as agricultural drainage water [23], textile wastewater [24], produced water [25], oily wastewater [26], and whey protein [27], both organic and inorganic materials have been widely investigated. Two-dimensional (2D) materials like MoS_2_, MXene [28,29], boron nitride [30], and graphene oxide were incorporated to augment the antifouling property and permeability using the hydrophilicity and capillary phenomenon of nanomaterials [15]. Furthermore, organic materials like carboxylic monoamines and covalent organic framework (COF) were used to enrich the nanofiltration performance in terms of permeability and protein repulse by tuning polyamide (PA) layer and sieving size control, respectively [4,31]. However, the alteration in PA structure led to significant loss in rejection, and the affinity between inorganic material and polymeric membrane decreased leading to significant loss in stability. Further investigation is required in this aspect to improve the stability and rejection by tuning the structural changes in PA.

In recent years, the zwitterionic polymers have become a promising material in membrane research for development of a universal antifouling and antibacterial membrane [32,33,34,35]. Both the cationic and anionic moieties of the zwitterionic materials on the membrane surface can be used to control the superhydrophilicity, biocompatibility, and non/fouling ability [36]. Since the ion pairs can trap significant amount of free water molecule by electrostatic interactions, a denser and tighter hydration layer can be formed on the membrane surface layer [37]. Several types of zwitterionic copolymers in this aspect have been synthesized and investigated to adapt different surface chemistry and application [33]. An et al. reported the homemade zwitterionic monomer N-aminoethyl piperazine propane sulfonate (AEPPS) incorporated nanofiltration membrane with improved water flux (84%) and rejection (~97%) with K_2_SO_4_ in the feed stream [5]. Interestingly, the incorporation of the zwitterionic materials also resulted in ~50% decrease in terms of bacterial (*S. epidermidis*) adhesion on the membrane surface. These observations further confirm that the zwitterionic material plays an important role in significantly enhancing universal nanofiltration performance either separation behavior or anti-pollute ability. The charge distribution and charge neutrality by optimization of parameters like charge density and the length of chain are important factor to control the overall antifouling behavior of the zwitterion-based polymeric membranes [38].

In the scope of the present study, an antifouling/antibacterial TFC NF membrane was fabricated using fast second interfacial polymerization. The PEI-SBMA zwitterionic polymer was synthesized by Michael addition reaction. The anchor moiety –COCl of TMC monomer was used to react with the amine group on PEI-SBMA. Several physicochemical characterizations were used in detail to prove and understand the modification mechanism. The separation performance was determined using different salt solutions (Na_2_SO_4_, NaCl, MgCl_2_) and their static antibacterial ability was evaluated using *E. coli* adhesion test for 24 h. Afterward, the dynamic fouling test was revealed by model charged foulants like BSA and lysozyme, respectively. This study provides a promising approach to fabricate a high-performance NF membrane which owns dense hydration layer to resist foulants approaching to the surface and maintains selectivity layer to avoid rejection loss. Also, this approach could be easily integral with existed NF membrane producing process.

## 2. Materials and Methods 

### 2.1. Materials

Powdered polyacrylonitrile (PAN) was supplied by Tong-Hwa Synthetic Fiber Co. Ltd. (Hsinchu, Taiwan). 1,3,5-benzenetricarbonyl chloride (TMC, 98%), 1-Methyl-2-pyrrolidinone (NMP, ACS grade), and *n*-hexane (HPLC grade) were purchased from Fisher Scientific (Pittsburgh, PA, USA). Phosphate-buffered saline (PBS, Biotechnology grade) tablets and diamine monomer piperazine (PIP, 98%) were purchased from VWR (Atlanta, GA, USA). Bovine serum albumin (BSA, 98%) was supplied by Lee BioSolutions (Maryland Heights, MO, USA). All inorganic salts (NaCl, MgCl_2_, and NaSO_4_) (purity 99%), polyethylenimine (PEI), sulfobetaine methacrylate (SBMA, 95%), and Lysozyme (LYZ, 98%) were procured from Sigma-Aldrich (St. Louis, MO, USA). Polyester non-woven was obtained from Membrane Science Inc. (Hsinchu, Taiwan). DI water with a conductivity value of 18.2 MΩ was used in all experiments.

### 2.2. Fabrication of the TFC NF Membranes

The zwitterionic polymer PEI-SBMA was synthesized by Michael addition reaction. In a typical synthesis, 1.955 g of SBMA monomer was added into 2 wt % of PEI/water solution in a round bottom flask. The reaction temperature was maintained at 95 °C in an oil bath for 6 h; afterward, the solution was cooled down to room temperature for 1 h. As a suitable nonsolvent was not available for purification of the product, the resultant product was purified by 1 kDa dialysis tube for 3 days, followed by freeze-drying. The synthesized PEI–SBMA was stored in vacuum chamber until used. The polymer was characterized using a Bruker 500 MHz NMR Spectrometer using D_2_O as solvent and reported in our early literature [32].

**Figure 1:**
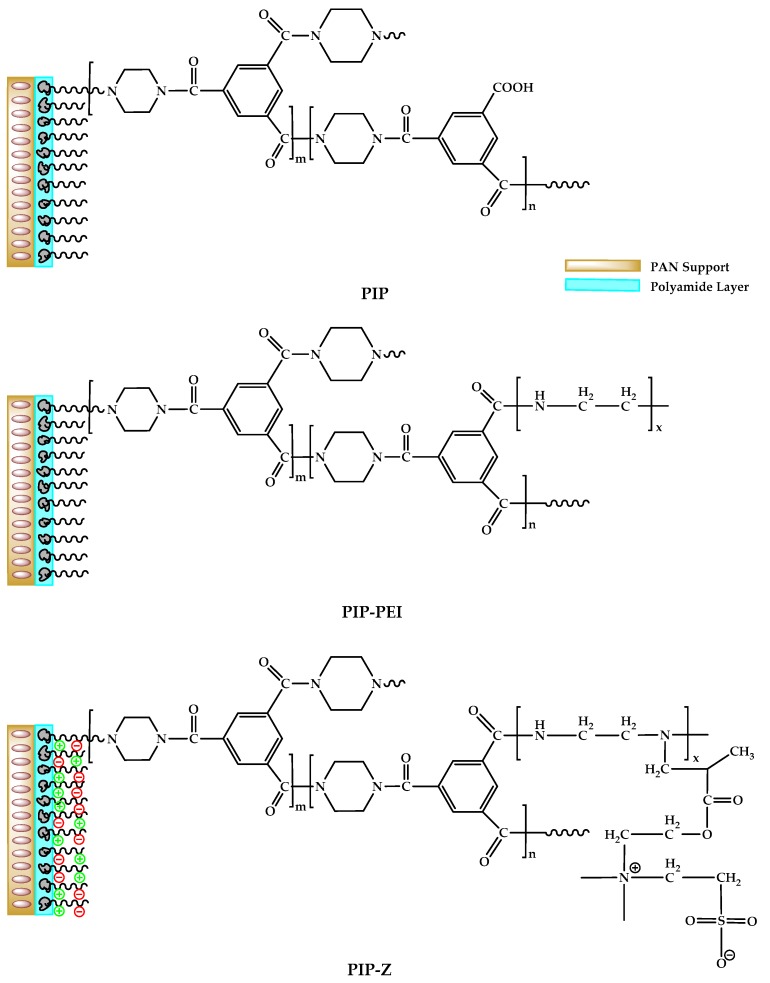
Chemical structure of the resultant membrane after second interfacial polymerization.

Wet phase inversion method was employed for fabrication of the support UF membrane. Commonly used polyester non-woven was placed on a clean glass plate for enhancement of the membrane mechanical property during the fabrication process. Thereafter, the casting solution comprised of 15 wt % PAN/NMP was spread on the non-woven by a 200 µm casting knife. The PAN UF membrane was rinsed with DI water several times and stored in DI water bath to remove residual solvent from the membrane. Prior to the fabrication of the TFC NF membrane, the PAN support membrane was washed with DI water three times. The support membrane was attached on the glass plate and excess water drops were removed from the surface by rubber roller. A 0.35 wt % PIP solution was poured into a Buna N frame covering on the surface and allowed to stay for 3 min to fill the pores on the membrane [4]. Afterward, the excess solution was removed using rubber roller, and the membrane was quickly contacted with 0.2 wt % TMC/hexane for 1 min to form a polyamide (PA) layer [22]. The resultant membrane was designated as PIP. The SIP was performed followed by obtaining PIP membrane. Residual amount of –COOCl on the PIP membrane was used to react with 1 wt % PEI/water and 1 wt % (PEI-SBMA)/water solution for a reaction time of 3 min, and subsequently denoted as PIP–PEI and PIP–Z, respectively. Finally, all these membranes were annealed at 70 °C inside hot air oven for 5 min and stored in water bath prior to the use. The chemical structure of the resultant membrane is shown in Figure 1.

### 2.3. Characterization of TFC NF Membranes

All the membranes were washed with DI water, and subsequently, dried in vacuum chamber prior to the characterization experiments. The surface functionality of the membranes was analyzed using attenuated total reflectance–Fourier transform infrared spectroscopy (ATR–FTIR, Pike Technologies, Madison, WI, USA). X-ray photoelectron spectrometry (XPS, Thermo Fisher Scientific Inc., Waltham, MA, USA) was conducted to characterize the chemical composition of the membranes. Membrane hydrophilicity was determined using water contact angle instrument (model OCA15EC, Future Digital Scientific, Garden City, NY, USA). Surface roughness was measured through atomic force microscope (AFM, Bruker, Billerica, MA, USA). Membrane morphology was investigated using field emission scanning electron microscope (FESEM S-4800, Hitachi Co., Tokyo, Japan). The surface charged of the membranes was assessed using SurPASS Electrokinetic Analyzer (Anton Paar, Ashland, VA, USA). The bacterial attachment on the membrane surface was monitored using confocal microscopy (LSCM A1R, Nikon, Tokyo, Japan).

### 2.4. Performance of the TFC NF Membranes

The membrane performance, water permeability in terms of flux (*J*) and salt rejection (*R*) were determined using the custom-made laboratory-scale cross flow NF apparatus. A detailed description of the apparatus has been provided in our early work [4]. The effective membrane area was 12 cm^2^ in the cell used for the analysis. The membranes were pre-compacted at 6.5 bar for 3 h to obtain a steady water flux. Furthermore, hydraulic pressure was reduced to certain pressure, and the flow rate was fixed at 0.6 LPM. The flux was recorded by a digital balance, and the rejection was monitored by a conductivity meter (Cond 3310, WTW, Germany). The flux J and rejection R were calculated using Equations (1) and (2), respectively.

(1) $$Flux\;\left(J\right)=\frac{g}{\rho \times A\times time}$$

(2) $$Rejection,\;R\%=\left(1-\frac{C_{p}}{C_{f}}\right)\times 100\%$$

where *g* was the mass of permeate collected after fixed time interval with fixed effective membrane area A. ρ is the density of water (1 kg/L). $C_{p}$ and $C_{f}$ were the concentration of the feed and permeate in the tank, respectively. All these measurements were performed at an operational temperature of 25 °C.

### 2.5. Bacterial Attachment

The bacterial attachment test was performed using Green fluorescent protein-*Escherichia coli* (GFP-*E. coli*) purchased from American Type Culture Collection (ATCC) to evaluate the antimicrobial behavior of the fabricated TFC nanofiltration membrane. In a typical experiment, 5.0 mg/mL peptone and 3.0 mg/mL beef extract were employed as the medium to culture the GFP-*E. coli*. Subsequently, the culture medium was incubated at 37 °C, and shaken at 100 rpm for 12 h until a stationary state with final bacteria concentration of 10^6^ cells/mL was achieved. The TFC nanofiltration membrane samples were washed with DI water and PBS buffer three times, respectively. Thereafter, the bacterial broth was placed on the microplate for 24 h. After the bacterial adhesion test, the membranes were removed and rinsed with PBS buffer three times to remove the loosely adhering bio-foulants. Finally, the membrane sample were placed on the sample stage of a confocal microscope with the excitation and emission wavelength of 488 and 520 nm, respectively, to evaluate bacterial attachment level on the membrane surface.

### 2.6. Dynamic Fouling Experiment

Dynamic antifouling test was performed using model protein solutions. In a typical measurement, the solution of model protein, BSA and Lysozyme, was used as a model foulant solution for such dynamic fouling test, respectively. Na_2_SO_4_ (1000 ppm) was dissolved in DI water solution followed by adding BSA (0.1 g/L). The fouling filtration was cycled 2.5 times. First, the DI water was used to compact the membrane at 6.5 bar for 3 h to steady the membrane. Afterward, Na_2_SO_4_ solution was applied at 6 bar for 1 h to obtain initial flux, $J_{0}$, then, the feed inlet was changed to Na_2_SO_4_ BSA solution tank for 5 h at bar to gain permeate flux, $J_{p}$. The washing steps were rinsing with DI water for 15 min at 1 bar followed by 15 min at 6 bars. The normalized flux, $J_{nor}$, was estimated using Equation (3).

(3) $$J_{nor}=\frac{J_{p}}{J_{0}}\times 100\%$$

## 3. Results

### 3.1. Physicochemical Properties of the TFC NF Membrane

**Figure 2:**
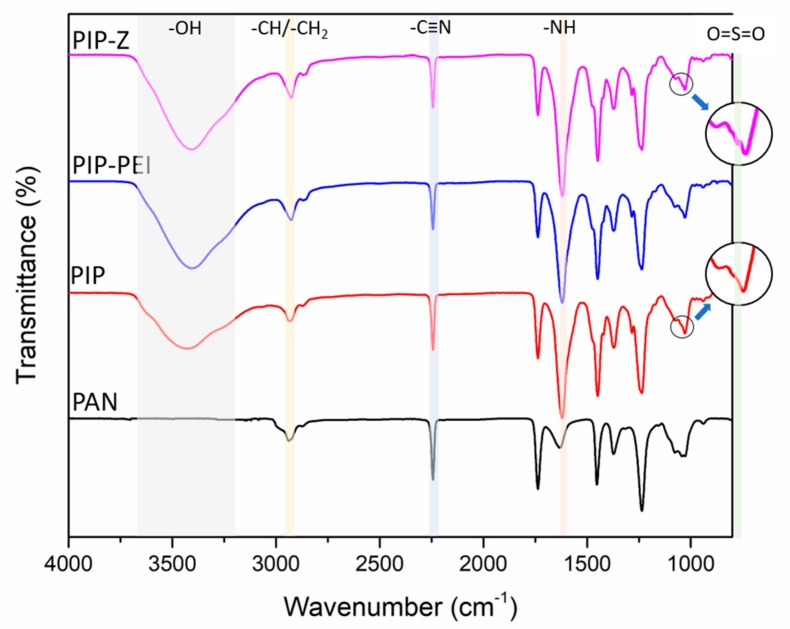
ATR-FTIR images of the membrane during each step of surface modification.

The reported zwitterionic TFC nanofiltration membrane was fabricated from the support membrane using a typical interfacial polymerization modification followed by second interfacial polymerization. Several characterization methods were employed to confirm the successful modification steps. The surface functional groups of supported PAN, PIP, PIP–PEI, and PIP–Z were revealed using ATR-FTIR spectral data as shown in Figure 2. In this study, the support PAN membrane was not hydrolyzed using NaOH solution prior to the use as it was reported in previous studies that hydrolysis led to significant decrease in pore size [39]. As evident from Figure 2, the characteristic peaks for support PAN membrane were observed with stretching vibration of the –CH and –CH_2_ group (2921 cm^−1^), stretching –C≡N (2242 cm^−1^) and vibration –CH (1451 cm^−1^), respectively [40,41]. The additional characteristic peak at 1623 cm^−1^ on all TFC membranes was assigned to amide I bonds, which verified the successful deposition of polyamide layer on the support membrane. Moreover, the broad peak in the range of 3200–3600 cm^−1^ was assigned to carboxyl group –OH which appeared due to conversion of the unreacted chloride acidic group on the TMC. In case of PIP-Z, an additional peak at 1042 cm^−1^ appeared, which was assigned to the stretching vibration of sulfonic acid groups (O=S=O) [18,20]. These observations proved that polyamide layer was successfully created on all the TFC membrane. Furthermore, the ATR-FTIR data also indicated that the PEI–SBMA was successfully grafted on to the PIP via second interfacial polymerization. The ATR-FTIR spectrum of PIP–PEI was almost identical with unmodified PIP due to a similar chemical bond (–CN) on the PEI polymer. Thus, XPS analysis was used to further confirm the successful second interfacial polymerization step.

**Figure 3:**
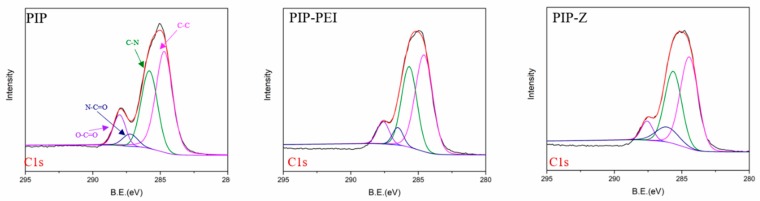
C1s photoelectron spectra of TFC membranes, PIP, PIP-PEI, and PIP-Z; where C–C, C–N, N–C=O, O–C=O peak at 284.6 eV, 285.6 eV, 287.5 eV, 288.5 eV, respectively.

The elemental analysis data obtained from XPS results is shown in Table 1. In case of, PIP the O/N ratio of PA layer was obtained to be 1.473 which was further used to determine the crosslinking degree of PA as 42.6% [42]. Moreover, the N element of PIP-PEI was obtained to be higher than the unmodified PIP membrane, which could be attributed to the successful rich-amine polymer PEI grafting onto the PIP PA layer. Elemental analysis also showed that S was only present in case of PIP-Z which confirmed the successful grafting of zwitterionic layer. Moreover, the enhanced elemental N in PIP–Z was attributed to the PEI–SBMA layer in good agreement with our early study [32]. Furthermore, the C1s bond analysis (Figure 3, Table 2) was obtained using XPSPEAK41 and furthermore, used to understand the alteration in chemical bonding after modification. Around 50% component on all of them was occupied by C–C bond because of either aqueous monomer PIP and organic monomer TMC or polymer PEI and PEI-SBMA having C–C bond within the chemical structure. Higher C–N bond contribution was observed for both PIP–PEI and PIP–Z, which was attributed to PEI polymer participating in the reaction. Enhanced contribution for N–C=O bonds of PIP–PEI was observed with a value of 0.0782 as compared to 0.0488 for PIP after grafting PEI mostly due to linking of more amine groups to the rest of the chloride acid group of TMC after generation of PA layer. The contribution of O–C=O bond in case of PIP-PEI was estimated to be least, primarily due to grafting of the amine group of PEI polymer which led to covering of the PA layer. Additionally, the zwitterionic component, SBMA, was attached to the amine group on PEI using the Michael addition reaction which led to masking of the anime group on external surface and hence, more O–C=O on the PA layer was exposed. These observations from the XPS analysis results confirmed the participation of the PEI in the reaction which was not evident from the ATR-FTIR analysis. Furthermore, the presence of zwitterionic polymer, PEI-SBMA, was also evidenced in case of PIP–Z. All these confirmations in terms of chemical reaction mechanisms were further used to analyze the physical structure alteration as well.

**Figure 4:**
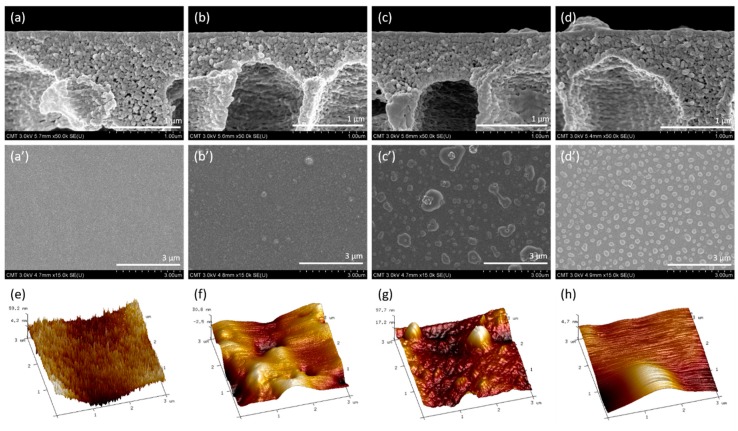
The morphology of surface/cross-section of TFC membranes, PAN support (**a**/**a′**), PIP (**b**/**b′**), PIP–PEI (**c**/**c′**), and PIP–Z (**d**/**d′**) as obtained from FESEM images and AFM images of PAN support (**e**), PIP (**f**), PIP–PEI (**g**), and PIP–Z (**h**).

The morphology of the surface and cross-section of TFC membrane was obtained using FESEM as shown in Figure 4a–d,a′–d′. The PAN support displayed a smooth surface, and no macropores were detected on the surface. The nodular PA structure was found in case of PIP which is additional evidence to verify the successful covering of PA layer on the support. The formation of nodular PA layer corresponds to the successful reaction of PIP and TMC as also reported in earlier studies [4,5,43]. Furthermore, a non-uniform surface was observed in case of PIP-PEI where the non-uniformity appeared after the second interfacial polymerization. As evident from ATR-FTIR and XPS analysis, the excess amount of unreacted amine groups on the PEI reacts with acid chloride groups which leads to disruption of the nodular shape and non-uniformity appears on the surface. Interestingly, the uniform nodular like tree clump was observed in case of PIP–Z, mostly due to specific linking of amine on PEI–SBMA to the anchor moiety (acid chloride) on the PIP. The thickness of the PA layer (~70 nm) was found to be unaffected even after the second interfacial polymerization for both the PIP–PEI and PIP–Z membrane [44]. The observed value of thickness was found to be similar to the earlier report [4]. PA layer thickness was unaltered mainly due to the peculiar method used for modification where only the residual acid chloride groups were reacted instead of reinitiating in the first interfacial polymerization [20]. Furthermore, AFM images of the TFC membranes were recorded as shown in Figure 4e–h. Table 3 shows all the different types of roughness parameters as obtained from AFM analysis. In the micro aspect, the uniform jagged structure was observed on PAN surface as shown in Figure 4e. The peculiar morphology generates due to a phase inversion step where solvent is replaced with non-solvent. After the formation of PA layer, smoother surface was observed as evident from lower roughness value of 7.58 nm. However, more nodular shapes were found probably due to covering of the pore by the PA layer. The irregular nodular structure was produced on the PIP–PEI, and the highest roughness value of 23.6 nm was observed on PIP–Z which was associated with tree clump structure. Earlier studies have mentioned that the membrane structure with high roughness value is able to improve the water flux [4]. Combining the chemical and morphology analysis, the modification chemistry and alteration in physical structure were verified before and after the modification. The membrane surface properties were further determined by water contact angle and zeta potential.

**Figure 5:**
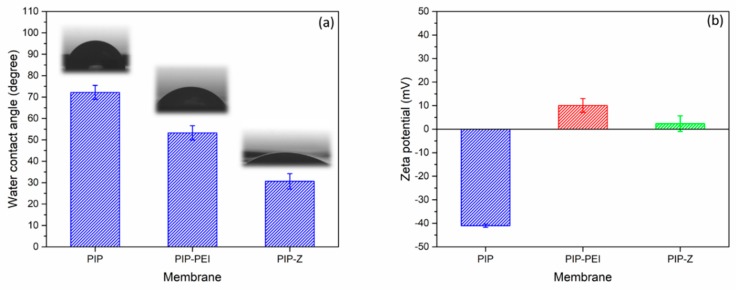
Water contact angle (**a**) and zeta potential (**b**) of TFC NF membrane.

Figure 5 indicates surface hydrophilicity and zeta potential results as obtained for the TFC membranes. The water contact angle is one of the commonly used methods to define the surface hydrophilicity in the membrane field [41]. The contact angle results could be significantly affected by chemical functional groups and surface morphology. Thus, the chemical and physical properties must be discussed together. The water droplet was placed on the PIP surface, and the water contact angle was estimated based on the Wenzel equation [45]. The PIP has the lowest average roughness (7.58 nm), and the corresponding water contact angle was obtained to be ~70° which was higher than the PIP–PEI surface. After grafting of the PEI, the contact angle decreased to 55°, which was not only due to higher roughness, but also higher charged density attributed to PEI polymer. The zwitterionic ion pair based nanofiltration membrane, PIP–Z, demonstrated the lowest contact angle ~30° probably due to the formation of hydration layer on the membrane surface. It was advantageous to create super hydrophilicity on the membrane surface and antifouling properties in presence of the zwitterions. Furthermore, to determine the alteration in surface charges during each step, the zeta potential was used to evaluate the charge density on different TFC membranes. Neutral environment with pH 7 was maintained for comparison of the surface charges on the different membranes. PIP membrane exhibited negative charge of −40 mV which was in good agreement with our early work [7]. The negative charge on the surface was contributed by –COOH groups converted from unreacted acid chloride on TMC. Even at neutral pH, the –COOH groups dissociate in solution in the form of –COO^−^ resulting in highly negative zeta potential value. Once the PEI was grafted, the zeta potential became positive with a value of +10 mV, which was associated with rich amine groups of PEI. The coating with amine groups masked the negative charge contribution from the –COOH groups and subsequent protonation of amine groups result in positive zeta potential value. PIP-Z displayed neutral charge close to zero due to the inherent ion pair feature of zwitterions on the surface [7]. Moreover, the shadowing effect exerted by the electrically neutral zwitterionic part on PIP–Z suppresses the positive charge contribution of the amine groups on PEI moiety.

### 3.2. Membrane Seperation Performance

**Figure 6:**
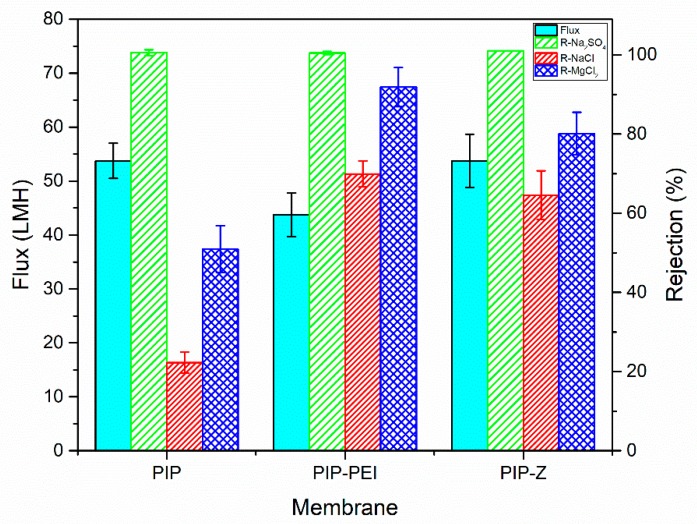
Water flux data and salt rejection as obtained for the different nanofiltration membranes. The operation pressure was fixed at 6 bar, and concentration of salt solution was 1000 ppm.

The membrane separation performance for different types of salt was recorded as shown in Figure 6. At neutral pH, the water flux of PIP was observed to be 53.7 L/m^2^ h, which was similar to PIP–Z. However, water flux in case of the PIP-PEI was observed to be slightly lower (43.75 L/m^2^ h) due to the formation of a non-uniform nodular structure on the PA layer in presence of PEI, which enhanced the mass transfer resistant. As evident from physical characterization methods, the tree clump structure did not fully cover the PA layer and the ion pair of a zwitterionic group form a hydrophilic hydration layer as well in case of PIP–Z. The rejection of PIP at neutral charge followed the order NaSO_4_ > MgCl_2_ > NaCl, which was in line with the Donnan exclusion theory [4,8]. However, the hydration radius of SO_4_^2−^ (0.379 nm) was larger than the other salt used in this study. Hence, its rejection for all the membranes, PIP, PIP–PEI, and PIP–Z, was found to be higher than 99%, as it was dominated by size exclusion. The divalent cationic Mg^2+^ was attracted by negatively charged surface, and consequently, the rejection of anionic counterpart was lower. The PIP–PEI exhibited the highest rejection of 91.8% when MgCl_2_ was used in the feed. The positively charged PEI moiety on the membrane repels the cationic Mg^2+^ species. Interestingly, when the surface charge was close to neutral, the rejection of MgCl_2_ and NaCl were 80.14% and 64.55%, respectively. Consequently, the rejection was found to be higher than the unmodified membrane, PIP and water permeability was observed to be similar. Additionally, the result was compared with the other literatures reported PIP-TMC based NF membrane in Table 4. PIP–Z has similar or higher water permeability as most of the other membrane, but the rejection of Na_2_SO_4_ and NaCl was relative higher than the other. It could be verified the resulting membrane not only maintain the required water flux but also increasing the salt rejection. Thus, the PIP–Z is a promising NF membrane for various water treatment applications in the future. 

### 3.3. Antifouling and Antibacterial Behavior

**Figure 7:**
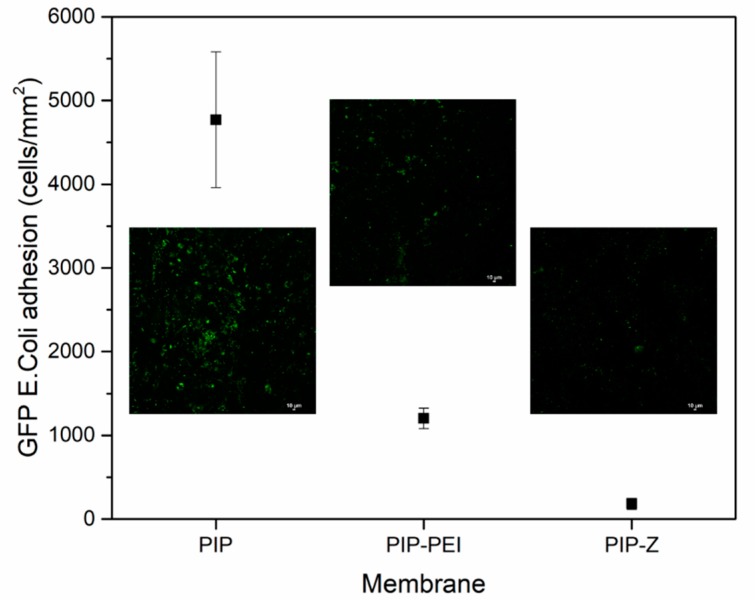
Confocal microscopic images of the TFC nanofiltration membranes after incubation using GFP-*E. coli* solution for 24 h as obtained during bacterial attachment test.

The TFC nanofiltration membranes used in this study were subjected to evaluation of antibacterial properties using GFP-*E. coli* as a model biofoulant as per the previous literature [52,53,54]. Figure 7 shows the confocal microscopic images of a gram-negative bacteria, GFP-*E. coli* adhesion on the different membrane surfaces. The outstanding antibacterial property for PEI–SBMA was illustrated in previous studies using a coating modification [32]. This work includes the use of the second interfacial polymerization to graft the zwitterionic moiety on the PA layer. The membrane was incubated in *E. coli* solution for 24 h, and thereafter, subjected to the quantitative evaluation as illustrated in Figure 7. The unmodified PIP (4771 cell/mm^2^) exhibited higher adhesion ability than PIP–PEI (1204 cell/mm^2^) and PIP–Z (183 cell/mm^2^), which could be contributed by superhydrophilic ‘ion pair’ moiety of PEI-SBMA. The superior antibacterial ability was also enhanced by the use of second interfacial polymerization as revealed by the higher adhesion ability value.

**Figure 8:**
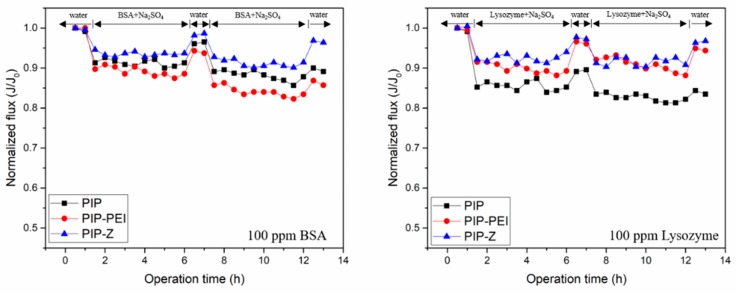
Dynamic fouling experiments with 100 ppm BSA and 100 ppm lysozyme. The pressure was maintained at 6 bar during fouling test.

Apart from a static bacterial adhesion test, a dynamic fouling experiment was performed using model foulant BSA and lysozyme, respectively. Figure 8 illustrates the performance of PIP, PIP-PEI, and PIP-Z membranes during the dynamic fouling experiments using both 100 ppm BSA and 100 ppm Lysozyme solutions separately as model protein foulants along with 1000 ppm Na_2_SO_4_ at pH 7 for an operational time up to 14 h. As evident from Figure 8, the normalized flux for all the membranes varied in the range of 1.0–0.8 for both the model protein foulants. The isoelectric point (pI) of BSA and lysozyme is 4.7 and 11.0, individually [55]. The positively charged PIP–PEI membrane exhibited lower normalized flux for the negatively charged BSA as compared to the PIP membranes. However, the normalized flux was found to be significantly improved in case of the zwitterionic ion-pair based neutrally charged PIP–Z membranes which confirm the improvement in terms of antifouling behavior due to peculiar modification step. In case of positively charged lysozyme, the modification using second interfacial polymerization led to significant improvement in normalized water flux as evident from higher flux for both PIP–PEI and PIP–Z as compared to PIP for longer operational time. The ion-pair zwitterion is able to bind with a lots of water molecular to reduce the charge foulant approaching the surface and enhance the surface hydrophilicity [5]. PIP-Z demonstrated the highest flux during operation; besides, the foulants can be easily cleaned from the membrane surface. Overall, PIP–Z was obtained to be a promising TFC nanofiltration membrane for various water treatment, especially, for a rich-biofoulant containing feed stream.

## 4. Conclusions

In this study, TFC nanofiltration membranes were fabricated using PAN support membranes through superfast second interfacial polymerization process involving PA layer incorporation followed by augmentation of zwitterionic moieties. Chemical structural changes during stepwise modification steps were further verified using characterization techniques like ATR-FTIR spectroscopy and XPS analysis. Morphology of surface as well as cross-section of the TFC nanofiltration membranes was investigated using FESEM images and the surface roughness was determined using AFM images. Combined chemical and physical characterization methods confirm the successful incorporation of PEI layer leading to non-uniform nodular shape and reappearance of uniformity on the membrane surface upon zwitterion augmentation. The ion-pair interaction in zwitterion-augmented PIP–Z nanofiltration membranes as compared to PIP membranes resulted in significant lowering of water contact angle from ~70° to ~30° and zeta potential from −40 mV to neutral. Average surface roughness increased significantly from 7.58 nm in case of PIP membranes to 15.8 nm and 23.6 nm in case of PIP–PEI and PIP–Z, respectively, during modification using second interfacial polymerization. Water permeability decreased significantly upon modification with PEI (43.75 L m^−2^ h^−1^) only leading to non-uniformity on the membrane surface. However, the membrane performance in terms of water permeability (53.75 L m^−2^ h^−1^) and salt rejection was significantly improved for zwitterion augmented PIP-Z membranes mainly due to neutral surface charges and improvement in surface morphology. Dynamic fouling tests using model protein foulants like BSA and LYZ showed that the antifouling property improved significantly in presence of zwitterionic SBMA species on PIP–Z membranes. Zwitterion-augmented TFC nanofiltration membrane, PIP-Z, exhibited lower bacterial attachment (183 cells/mm^2^) than the unmodified membrane (4771 cells/mm^2^) using GFP *E. coli* due to the presence of ‘ion pair’ and enhanced antifouling properties due to the use of second interfacial polymerization step during modification. Overall, the fabricated TFC nanofiltration membranes using second interfacial polymerization modification steps exhibited excellent performance in terms of water flux with enhanced rejection of the monovalent as well as divalent salts. Unlike conventional in situ modification, the SIP method used in this study was able to avoid the influence on the structure of the selective layer and additionally, promoted the antifouling and antibacterial properties. With inherent advantages of low-cost nanofiltration processes, these TFC nanofiltration membranes have huge commercial prospect for low-cost production of clean water and wastewater pretreatment for pollution reduction.

## Figures and Tables

**Table 1:** Analysis of the TFC membranes.

	C1s (%)	N1s (%)	O1s (%)	S2p (%)
PIP	74.59	10.27	15.13	-
PIP-PEI	72.68	13	14.31	-
PIP-Z	70.74	12.72	15.92	0.62

**Table 2:** Bond analysis of the TFC membranes.

	C–C	C–N	N–C=O	O–C=O
PIP	0.5056	0.3438	0.0488	0.1017
PIP-PEI	0.5076	0.3602	0.0782	0.0542
PIP-Z	0.4705	0.3740	0.0613	0.0941

**Table 3:** Value as obtained from AFM image analysis.

	R_a_ (nm)	Rq (nm)	Rmax (nm)
PAN support	12.2	15.4	128
PIP	7.58	9.41	54.7
PIP-PEI	15.1	20.2	133
PIP-Z	23.6	35.7	225

**Table 4:** Comparison the other literature reported 349 PIP–TMC based NF membrane.

Membrane/Support	Water Permeability (L m^−2^ h^−1^ bar^−1^)	R%(Na_2_SO_4_)/Concentration (ppm)	R%(NaCl)/Concentration (ppm)	Operating Pressure (bar)	Reference
PIP-Z/PAN ^a^	8.6	99/1000	64.55/1000	6	This study
PIP+AEPPS/PSf ^b^	7.7	-	30/1000	6	[5]
PIP−AEPPS/PAN ^a^	9.5	98/1000	45/1000	6	[46]
PIP+CTAB/PES ^b^	4.9	90/1000	70/1000	10	[47]
PIP+PVA/PSf ^b^	10.6	-	16/3000	7	[48]
PIP+biogenic Ag+/PSf ^b^	5.0	86.2/2000	-	3.5	[49]
PIP+ammonium salts/PES ^b^	8	-	46/500	3.5	[50]
NF270(polyamide)/PSF	11.6	94/2000	51/2000	10	[51]

^a^ second interfacial polymerization. ^b^ In-situ modification.
